# A New Symptom in Scarlet Fever

**Published:** 1911-05

**Authors:** 


### A New Symptom in Scarlet Fever.

In the February number of the *Archives de médicine des enfants,* Pastia, of Bucharest, describes a new sign in scarlet fever which, if it is of constant occurrence, will prove of as great diagnostic value as Koplik's spots in measles. This symptom consists of transverse lines, usually two or three, in the fold of the elbow. They are of a rose hed hue at first, but in a few days turn red or wine colored. In the severe cases they are ecchymotic. Between the lines the erythematous eruption characteristic of scarlatina is seen. Similar lines have been observed in the axilla in a few cases, but they are of less intense color and of shorter duration in that situation.

These lines are visible before the appearance of the rash, remain throughout the eruptive stage, and persist as lines of pigmentation after desquamation is completed. Pastia found this symptom present in 94 per cent of the cases of scarlet fever in the hospital for contagious diseases in Bucharest. As far as his experience goes, it is absent in the other exanthemata and in all forms of drug rash. If the danger of contagion is greatest during the tonsillar stage of the disease and not during the period of desquamation, as was formerly thought, any symptom which will aid in establishing the diagnosis before the appearance of the rash will be of great value in preventing the spread of the infection.—*New York Medical Journal.*

